# Analysis of symptomatic brain aneurysms with three-dimensional aneurysm wall enhancement

**DOI:** 10.1007/s10143-025-04120-w

**Published:** 2026-01-24

**Authors:** Chengcheng Zhu, Elena Sagues, Bing Tian, Huilin Zhao, Andres Gudino, Navami Shenoy, Diego Ojeda, Carlos Dier, Arshaq Saleem, Linder Wendt, Sebastian Sanchez, Jacob M Miller, Luis Noboa, Katherine Guijarro-Falcon, Matthew T Jones, Randall Krug, Santiago Ortega-Gutierrez, David Hasan, Edgar A Samaniego

**Affiliations:** 1 Department of Radiology, University of Washington, Seattle, USA; 2 Department of Neurology, University of Iowa, 200 Hawkins Dr., Iowa City, IA 52242 USA; 3 Department of Radiology, Changhai Hospital, Shanghai, China; 4 Department of Radiology, Renji Hospital, Shanghai, China; 5 Department of Neurology, University of Connecticut, Hartford, CT USA; 6 Carver College of Medicine, University of Iowa, Iowa City, IA USA; 7 Institute for Clinical and Translational Science, University of Iowa, Iowa City, IA USA; 8 Department of Neurology, Yale University, New Haven, CT USA; 9 Department of Neurosurgery, University of Iowa, 200 Hawkins Dr., Iowa City, IA 52242 USA; 10 Department of Radiology, University of Iowa, 200 Hawkins Dr., Iowa City, IA 52242 USA; 11 Department of Neurosurgery, Duke University, Durham, NC USA

**Keywords:** Unruptured intracranial aneurysms, Vessel wall, Magnetic resonance imaging, Subarachnoid hemorrhage

## Abstract

To determine whether three-dimensional quantitative assessment of aneurysm wall enhancement (AWE) improves the identification of high-risk intracranial aneurysms (IAs) compared to existing risk scoring systems. This cross-sectional, multicenter observational study included patients with saccular IAs who prospectively underwent 3T high-resolution MRI between 2018 and 2023 at the University of Iowa, Changhai Hospital, and Ren Ji Hospital. IAs were classified as symptomatic if associated with rupture, sentinel headache, cranial nerve palsy, or neurological symptoms due to mass effect. AWE was quantified using three-dimensional aneurysm wall mapping. The diagnostic performance of the PHASES score was compared to a multivariate logistic regression model developed using all-subsets regression, incorporating PHASES components along with sex, smoking status, aneurysm morphology, and AWE data. A total of 334 patients with 387 saccular IAs were analyzed; 73 aneurysms (19%) were symptomatic. In multivariate analysis, three-dimensional circumferential AWE (3D-CAWE) was independently associated with symptomatic presentation (OR 6.1, 95% CI 2.1–19.5; *p* < 0.001). The PHASES score achieved an area under the curve (AUC) of 0.64 (95% CI 0.56–0.71) for discriminating symptomatic IAs. A comprehensive model including age, smoking status, size ratio, and 3D-CAWE significantly outperformed PHASES, achieving an AUC of 0.80 (95% CI 0.74–0.85; ΔAUC = 0.16, 95% CI 0.09–0.23; *p* < 0.001). A comprehensive risk model that integrates clinical variables, aneurysm morphological features, and 3D-CAWE significantly improves the classification of symptomatic IAs compared to the PHASES score alone.

**Supplementary Information:**

The online version contains supplementary material available at 10.1007/s10143-025-04120-w.

## Introduction

Intracranial aneurysms (IAs) are estimated to affect 5% of the total population and present an annual rupture risk of 1–2%.^1^ Clinical management of unruptured IAs requires balancing the risk of rupture against the procedural risks associated with intervention [2]. However, determining the risk of rupture remains challenging, as existing scoring systems have limited accuracy and are not validated across all populations [3]. Various novel neuroimaging techniques and biomarkers have been studied to improve the assessment of IAs. Aneurysm wall enhancement (AWE) on high-resolution magnetic resonance imaging (MRI) has emerged as a promising biomarker, associated with inflammation, wall remodeling, symptomatic presentation, and rupture risk [4–6]. However, the determination of AWE in these studies primarily relies on subjective visual inspection of two-dimensional multiplanar images. These methods of AWE assessment are susceptible to human error and bias. To overcome these limitations, a method for objectively quantifying AWE using three-dimensional (3D) aneurysm models has been developed [7]. In a previous study of 104 aneurysms, this 3D circumferential AWE (3D-CAWE) metric outperformed the PHASES (Population, Hypertension, Age, Size, Earlier subarachnoid hemorrhage, and Site) score in identifying symptomatic aneurysms [8]. In this multicenter study, we aim to evaluate whether integrating AWE metrics with established clinical risk factors and aneurysm morphological characteristics enhances the identification of symptomatic intracranial aneurysms.

## Methods

### Study population

This was a cross-sectional observational international multi-centric study. After institutional review board approval and data sharing agreement, patients with IAs from the University of Iowa Health Care in the United States, and from the Changhai and Ren Ji hospitals in China were included in this study in a consecutive order. Informed consent was waived at the University of Iowa because the data were originally collected for clinical purposes and the study involved minimal risk to participants. Informed consent was obtained at the Changai and Ren Ji hospitals prior to imaging. Patients were scanned prospectively using 3T high-resolution MRI between 2018 and 2023. Patient clinical information was anonymized prior to sharing high-resolution MRI imaging data for analysis at the University of Iowa. The study included patients with untreated saccular IAs larger than 2 mm, both unruptured and ruptured. Patients underwent imaging as part of their neurological assessment. In symptomatic cases, AWE was evaluated on the initial imaging before aneurysm securing. Exclusion criteria were: (1) poor image quality or motion artifacts that interfered with accurate co-registration of T1W1 and T1W1 + Gd sequences; (2) fusiform or thrombosed aneurysms; and (3) IAs located near the cavernous sinus, where the aneurysm wall boundaries could not be clearly visualized. Part of this cohort, 73 of the 334 patients, have been reported in a previous publication [9]; however, the current analysis combines data from a multicentric cohort to achieve more generalizable results. Additionally, a more refined quantification software was utilized in this study.

Clinical data was retrieved from medical records at each institution. The clinical variables collected included population, age, sex, hypertension, and smoking status. Aneurysm locations were classified as ICA, MCA, anterior cerebral artery or anterior communicating artery, posterior cerebral artery, posterior communicating artery or the ICA portion where the posterior communicating artery originates, basilar artery, superior cerebellar artery, anterior and posterior inferior cerebellar artery [10, 11]. Aneurysms in high-risk locations for rupture or symptom development include those in the anterior communicating artery, posterior communicating artery and the posterior circulation. The PHASES score was calculated for each patient [3]. 

Aneurysm morphological assessment was performed on 3D angiographic models, ensuring consistency by using the same dome-to-neck projection angle for all measurements. These included the aneurysm’s maximum diameter, neck diameter, maximum height, perpendicular height from the middle of the neck, and the average diameter of the parent vessel measured at 1.5 mm from the aneurysm neck [12]. In bifurcation aneurysms, the parent vessel and both daughter vessels were measured to calculate their average. Similarly, for aneurysms originating from the anterior communicating artery, the bilateral A1 and A2 segments of the anterior cerebral artery were measured. In cases of azygous anterior cerebral artery, the single A1 and both A2 segments were measured. Aneurysm size was defined as the aneurysm maximum diameter. The size ratio was calculated by dividing the maximum aneurysm height by the average parent vessel diameter [12]. The aspect ratio was calculated by dividing the perpendicular aneurysm height by the neck diameter [10, 12]. Irregular shape, defined by the presence of daughter sacs or blebs, was adjudicated by a senior investigator based on 3D angiographic reconstructions [13]. 

IAs were classified as symptomatic based on the following criteria: (1) ruptured at presentation, (2) cranial nerve neuropathy due to aneurysm mass effect or compression, (3) sentinel headache, defined as unusually severe and sudden headache in the past 2 weeks before presentation, and (4) presence of neurological symptoms due to mass effect in the brainstem [14–16]. A composite symptomatic outcome was defined as the presence of any of these criteria. This approach was based on the rationale that these clinical presentations share a common biological mechanism related to aneurysm wall instability and associated inflammatory changes. In patients with multiple aneurysms, only one IA was designated as symptomatic. This selection was based on clinical presentation and imaging findings.

### Imaging acquisition

The University of Iowa Health Care used a 3T MRI Siemens scanner (MAGNETOM, Skyra), the Changhai, and Ren Ji Hospitals used 3T MRI Siemens scanners (MAGNETOM, Prisma). The imaging protocol included: T1WI and T1WI + Gd obtained after the administration of 0.1mmol/kg Gd-DTPA-based contrast agent (Gadavist, Bayer Pharmaceuticals, Whippany, NJ, Table 1). Prior to analysis, the T1WI and T1WI + Gd images underwent isotropic resampling and rigid co-registration within 3D Slicer (version 5.2.1), as described in Fedorov et al. [17]. Images were resampled to an isotropic voxel size of 0.6 mm³, using linear interpolation to standardize voxel dimensions across all images. Rigid co-registration was applied to align each subject’s T1WI and T1WI + Gd sequences.

**Table 1:** Imaging parameters for the three cohorts 3T HR-MRI

	University of Iowa	Changai Hospital	Ren Ji Hospital
Model	Siemens MAGNETON Skyra	Siemens MAGNETOM Prisma	Siemens MAGNETOM Prisma
Acquired spatial resolution (mm)	0.6 × 0.6 × 0.6	0.5 × 0.5 × 0.5	0.6 × 0.6 × 0.6
Post contrast time (min)	5	2	2
Acquisition time of VWI (min)	3:29	8	9:13
Pulse sequence type	3D SPACE*	3D SPACE	3D SPACE
TR (msec)	900	900	1000
TE (msec)	15	5.6	15
FOV (mm)	200 × 200	256 × 160	220 × 195
Matrix (mm)	320 × 320	331 × 384	384 × 345
Slice thickness, mm	0.63	0.5	0.6
Turbo Factor	52	60	60

*3D SPACE: Sampling Perfection with Application-optimized Contrasts by using different flip angle Evolutions

### Image analysis

Image analysis was conducted at the University of Iowa by two investigators with two years of experience in aneurysm wall analysis using 3D Slicer (E.S. and A.G.). The investigators were blinded to the clinical data. In cases of disagreement, a senior investigator (E.A.S.) with over 10 years of experience adjudicated the area corresponding to the aneurysm lumen-wall boundary. Segmentations of the aneurysm sac and parent vessel were performed in 3D Slicer using T1WI + Gd sequences. Multiplanar views facilitated precise segmentation of the aneurysm lumen, with a segmentation voxel size of 1.08 mm³. An additional aneurysm wall segmentation was generated using the “Hollow” tool in 3D Slicer. This tool automatically creates a one-voxel-thick 3D segmentation based on the outer boundary of the aneurysm lumen. To exclude the neck region, the parent artery segmentation is subtracted from the wall segmentation. All segmentations were carefully reviewed to ensure accurate representation of the aneurysm wall without contamination from the lumen or adjacent structures, such as the cavernous sinus or pituitary stalk. Contaminated voxels were manually removed as needed. Cases were excluded if the wall boundaries could not be clearly visualized. Additionally, a label map of the corpus callosum was created for signal intensity normalization, due to its consistent contrast uptake, minimal enhancement, and reduced partial volume effects [18]. The aneurysm wall and corpus callosum segmentations were resampled into co-registered T1WI and T1WI + Gd images and exported as label maps for MATLAB analysis.

**Fig. 1:**
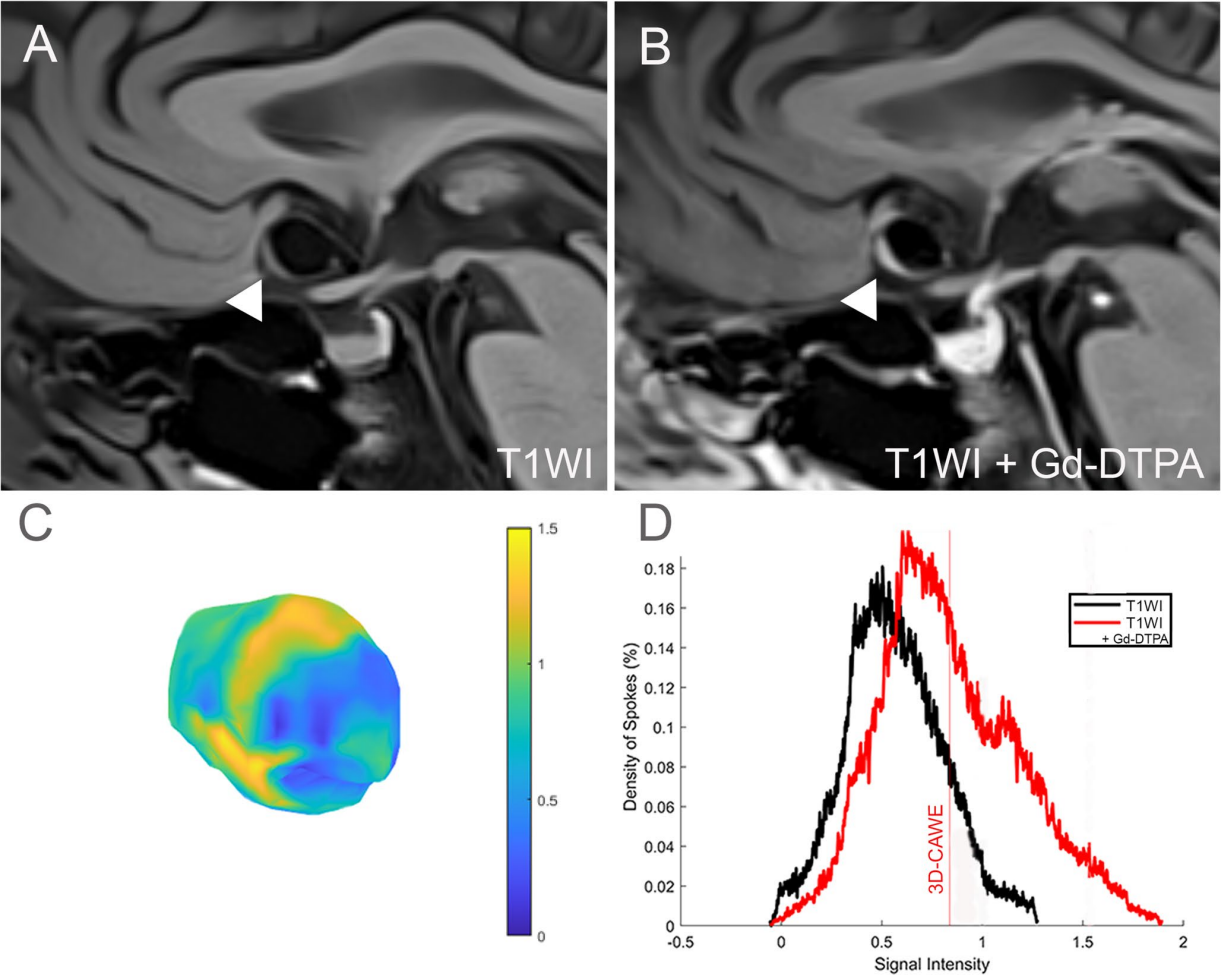
Three-dimensional colormap and histogram representation of AWE quantification. **A** T1WI high-resolution MRI and (**B**) T1WI + Gd high-resolution MRI of an anterior communicating artery aneurysm (white arrowheads). **C** Three-dimensional colormap of the T1WI + Gd signal intensity of the aneurysm wall, with areas of higher AWE shown in yellow and areas of lower AWE shown in blue. **D** Histogram representation of T1WI (black) and T1WI + Gd (red) signal intensity distribution. The curve is shifted to the right after contrast-Gd administration. The mean signal intensity in T1WI + Gd is calculated as 3D-CAWE (red line)

AWE quantification was performed using a customized MATLAB R2024a (MathWorks, Natick, MA) script that analyzes 3D aneurysm wall segmentations voxel-by-voxel to measure signal intensity. The script generates a metric representing the average of the 3D circumferential aneurysm wall enhancement (3D-CAWE). Results were visualized as 3D color maps and histograms (Fig. 1). While previously validated [9], the script was adapted for this larger cohort of aneurysms to address individual wall thickness variations, improving voxel selection accuracy over the orthogonal spokes sampling method. The script has also been refined to minimize flow artifacts using acquisition techniques such as “black blood” imaging [9]. AWE metrics, histograms, and color maps were compared with high-resolution MRI images for quality control. The Intraclass Correlation Coefficient (ICC) was calculated for the 3D-CAWE of 10 common cases segmented by each investigator (E.S. and A.G.). AWE measurements were flagged as abnormal under the following conditions: (1) the mean signal intensity on pre-contrast T1WI exceeded that of the post-contrast T1WI + Gd; (2) the color map showed high enhancement near known artifact-prone areas such as the cavernous sinus wall or pituitary stalk; or (3) areas of very low enhancement were detected in non-wall regions such as the aneurysm lumen, cerebrospinal fluid, or air within the cavernous sinus. When identified, these issues were addressed by repeating the co-registration of T1WI and T1WI + Gd, refining wall segmentation to exclude contaminating voxels, and reducing the automated shell thickness to avoid inclusion of external structures.

### Statistical analysis

Statistical analysis was performed using R, version 4.3.3. Continuous variables were represented with medians and interquartile range (IQR), while categorical data were represented with counts and percentages. Wilcoxon rank sum tests were used to compare continuous variables across strata, while Chi-square test was used for categorical comparisons. P-values less than 0.05 were considered statistically significant for all comparisons, and corrections for multiple comparisons were not employed due to the exploratory nature of the analysis. Logistic regression models were constructed to calculate the likelihood of an aneurysm being symptomatic. The models were selected using an all-subsets regression approach in which every possible combination of the candidate predictors was attempted and the model with the lowest Akaike Information Criterion was selected. Two models were developed: (1) PHASES score and (2) a comprehensive model combining clinical information, morphological aneurysm data, and AWE metrics. The performance of the models was evaluated using receiver operating curves, with areas under the curve (AUC), accuracy, sensitivity, and specificity reported. Sensitivity and specificity were determined using the Youden Index, which identifies the optimal threshold by maximizing the sum of sensitivity and specificity. The models’ performance was compared using the DeLong test, with the AUC difference and p values reported. A subgroup analysis was performed dividing the outcome into (1) unruptured (including sentinel headaches, cranial nerve palsy and mass effect symptoms) and (2) ruptured.

## Results

**Fig. 2:**
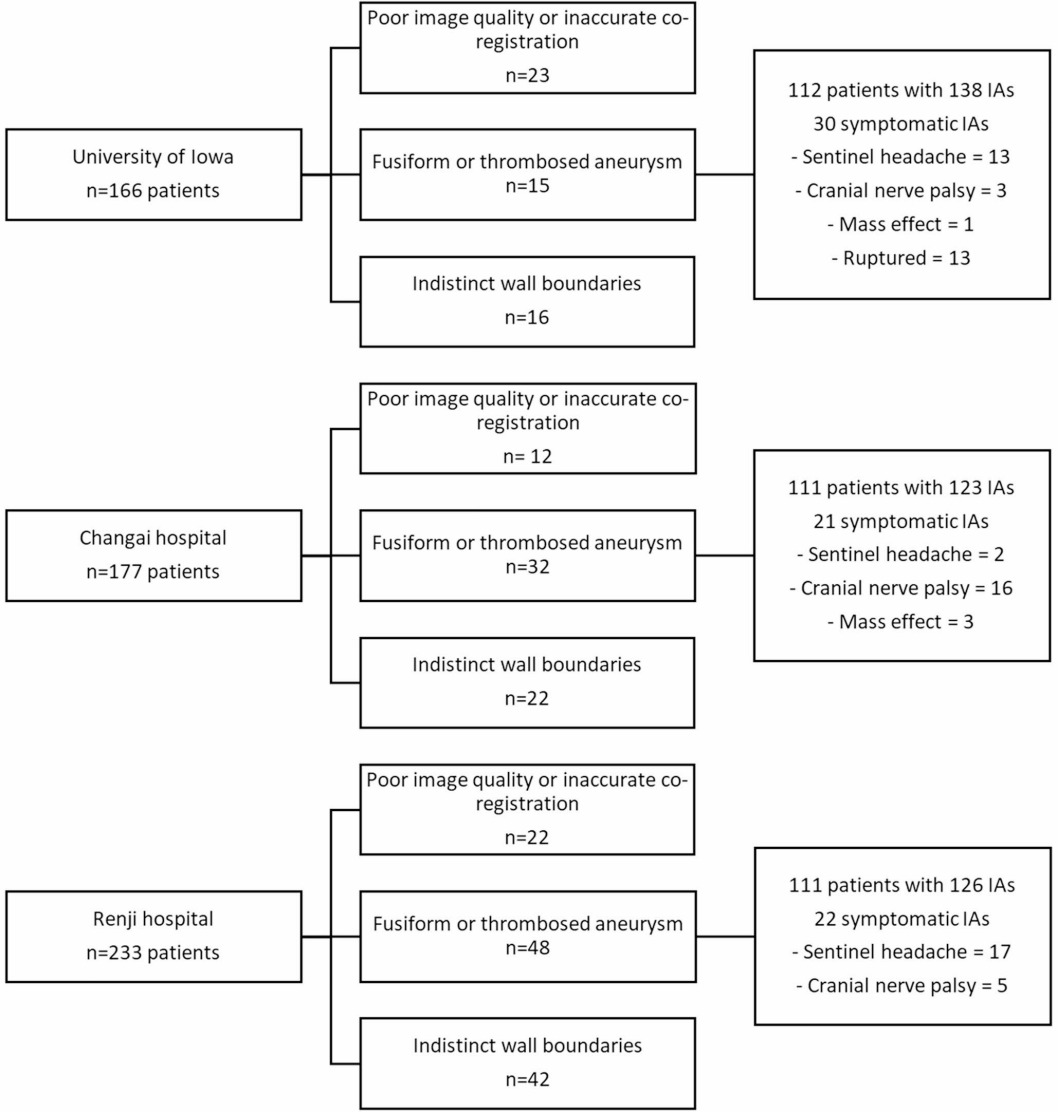
Population selection flow diagram

A total of 576 patients were screened, and 57 patients (9%) were excluded due to poor image quality, 95 (16%) with fusiform or thrombosed IAs, and 80 patients (14%) with IAs where the wall boundaries could not be clearly visualized. Ultimately, 334 patients with 387 IAs were included in the study: 139 (36%) from the University of Iowa Hospital, 123 (32%) from Changhai Hospital, and 126 (32%) from Renji Hospital (Fig. 2). Of the 387 included IAs, 73 (19%) were symptomatic at presentation. Among these symptomatic IAs, 13 (18%) were ruptured, 32 (44%) were associated with sentinel headaches, 24 (33%) caused cranial nerve palsies, and 4 (5%) presented with neurological symptoms due to mass effect. Symptomatic patients were younger than asymptomatic patients (median 59, IQR 52–66 versus median 64, IQR 53–71 years, *p* = 0.022, Table 2). Symptomatic IAs were larger than asymptomatic ones (median size of 8.40 mm, IQR 6.20–15.90 versus 5.50 mm, IQR 4.20–8.20, *p* < 0.001). PHASES score was higher in symptomatic patients compared to asymptomatic ones (median 6 IQR 4–8 vs. 4 IQR 2–6 vs., *p* < 0.001). Median 3D-CAWE values were similar across the Iowa, Changhai, and Renji cohorts (0.72, 0.71, and 0.70, respectively; *p* = 0.372). The ICC for the 3D-CAWE values between the two investigators for the 10 common segmentations was 0.82 (CI 0.46–0.95, *p* < 0.001), indicating good reliability. Symptomatic IAs had higher 3D-CAWE than asymptomatic IAs (median 0.86, IQR 0.67-1 versus median 0.69, IQR 0.57–0.86, *p* < 0.001).

**Table 2:** PHASES (Population, Hypertension, Age, Size, earlier SAH and site of aneurysm) and additional patient characteristics

Characteristic	Asymptomatic, *N* = 314^*1*^	Symptomatic, *N* = 73^*1*^	*p*-value^2^
Population			
North American	108 (34)	30 (41)	0.268
Chinese	206 (66)	43 (59)	0.268
Hypertension	174 (55)	39 (53)	0.826
Age	64 (53, 71)	59 (52, 66)	0.022
Age > 70 years	85 (27)	11 (15)	0.037
Size	5.50 (4.20, 8.20)	8.40 (6.20, 15.90)	< 0.001
< 7 mm	199 (63)	29 (40)	
7–9.9 mm	67 (21)	16 (22)	
10–19.9 mm	41 (13)	17 (23)	
≥ 20 mm	6 (2)	10 (14)	
Earlier SAH	0	0	NA
Aneurysm location			
ICA	134 (43)	31 (42)	0.953
MCA	61 (19)	13 (18)	0.790
ACOM, PCOM or posterior circulation^3^	119 (38)	28 (38)	0.876
Total PHASES score	4 (2, 6)	6 (4, 8)	< 0.001
Female sex	230 (73)	55 (75)	0.585
Current smoking	48 (15)	18 (25)	0.048
Irregular shape	65 (21)	20 (27)	0.191
3D-CAWE	0.69 (0.57, 0.86)	0.86 (0.67, 1.00)	< 0.001

^1^n (%); Median (Q1, Q3)

^2^Pearson’s Chi-squared test; Wilcoxon rank sum test

^3^High risk locations: ACOM, PCOM and posterior circulation.

Abbreviations: *ACOM*: anterior communicating artery, *PCOM*: Posterior communicating artery, *3D-CAWE*: three-dimensional circumferential aneurysm wall enhancement.

### Model performances

**Fig. 3:**
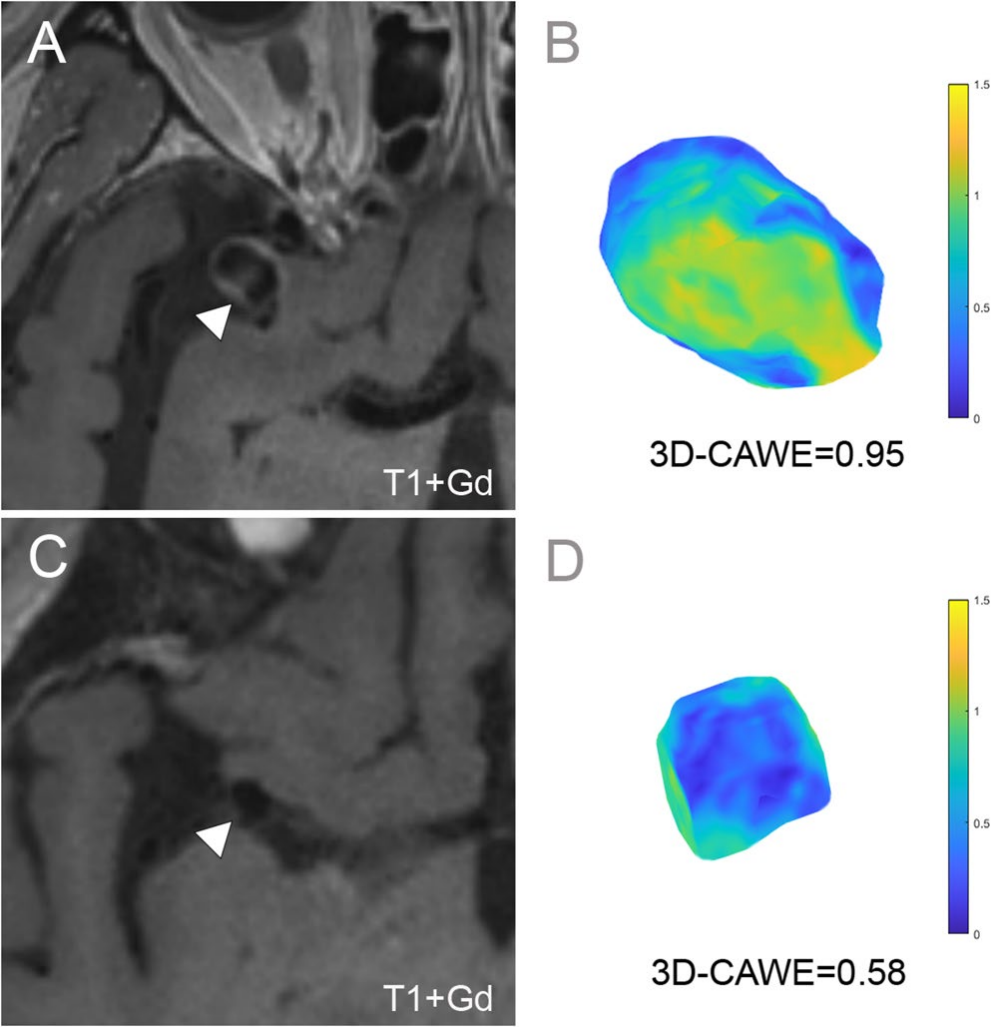
3D-CAWE is associated with IA symptomatic presentation. **A** T1WI + Gd high-resolution MRI of a right MCA IA (white arrowhead) presenting with sentinel headaches. **B** The 3D-CAWE colormap shows significant Gd enhancement (yellow). **C** T1+Gd high-resolution MRI of an asymptomatic right MCA aneurysm (white arrowhead). **D** 3D-CAWE does not show areas of increased Gd uptake (blue). The 3D-CAWE value was higher in the symptomatic IA (0.95) compared to the asymptomatic one (0.58)

**Fig. 4:**
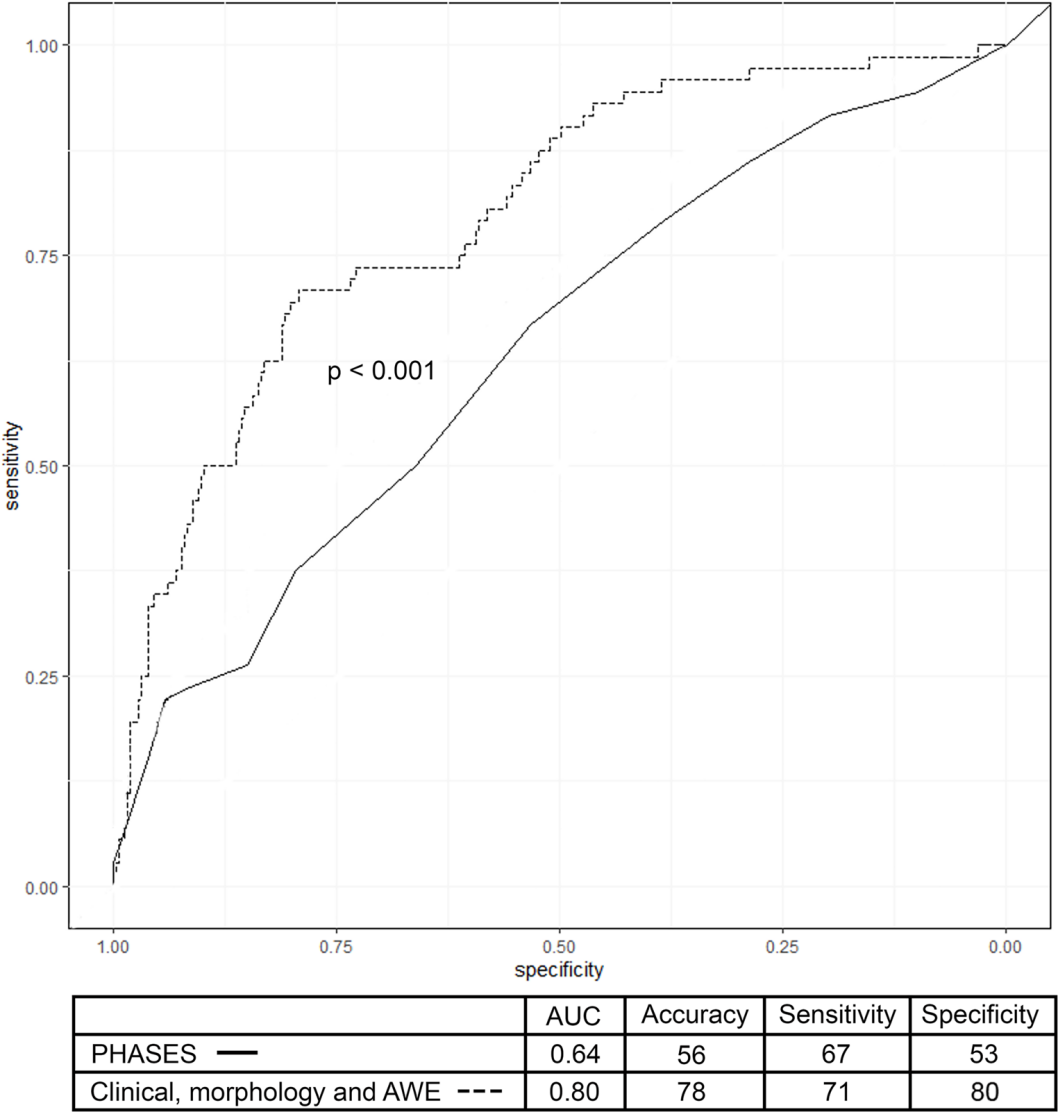
AUC of logistic regression models for classifying symptomatic aneurysm presentation. Three different models were created: 1) PHASES score, 2) PHASES score combined with AWE, and 3) a comprehensive model selected through all-subsets regression, and that included: age, smoking, size ratio, irregular shape, and AWE

The PHASES score achieved an AUC of 0.64 (CI 0.56–0.71), with 56% accuracy, 67% sensitivity, 53% specificity (Fig. 4). In multivariate analysis, age (OR 0.96, CI 0.93–0.98, *p* < 0.001), size ratio (OR 1.6, CI 1.3–1.8, *p* < 0.001) and 3D-CAWE (OR 6.1, CI 2.1–19.5, *p* < 0.001, Fig. 3) were independently associated with symptomatic presentation. The comprehensive model for estimation of symptomatic presentation, which included age, smoking, size ratio, and 3D-CAWE through all-subsets regression, achieved the highest performance with an AUC of 0.80 (CI 0.74–0.85), an accuracy of 78%, a sensitivity of 71%, a specificity of 80%, and an AIC of 319 (Table 3). The observed difference between the PHASES and this model AUC was − 0.16 (CI -0.23-0.85, *p* < 0.001). In all the top five models from the all-subsets regression, 3D-CAWE was independently associated with symptomatic presentation (supplementary Table 1).

**Table 3:** Multivariate logistic regression model for symptomatic aneurysm presentation

Characteristic^1^, *N* = 387	OR	95% CI	*p*-value
Age	0.96	0.93–0.98	< 0.001
Smoking	1.9	0.96–3.76	0.060
Size ratio	1.6	1.3, 1.8	< 0.001
3D-CAWE	6.1	2.1, 19.5	< 0.001

^1^Predictors were selected by all-subsets regression, AIC 319

Abbreviations: *3D-CAWE*: three-dimensional circumferential aneurysm wall enhancement.

### Subgroup analysis: unruptured versus ruptured IAs

Among unruptured cases, age (OR 0.96, CI 0.93–0.98, *p* < 0.001), size ratio (OR 1.7, CI 1.4–2.1, *p* < 0.001) and 3D-CAWE (OR 2.4, CI 0.7–7.3, *p* = 0.128) were selected in the final model through all-subsets regression (supplementary Table 2). In contrast, among ruptured cases, smoking (OR 8.7, CI 2.6–31.6, *p* < 0.001), irregular shape (OR 4, CI 1.1–14.3, *p* = 0.028), and 3D-CAWE (OR 10.5, CI 2.4–50.2, *p* = 0.002) were selected.

## Discussion

In this large international multi-center cohort of saccular IAs analyzed using 3D-CAWE mapping, the best performing model for estimating symptomatic IAs presentation combined clinical information, aneurysm morphology and AWE metrics. This model outperformed the PHASES score in estimating symptomatic IA presentation. Although PHASES was developed for predicting risk of rupture, it has been used extensively for risk stratification of unruptured IAs. Incorporating AWE into current scoring systems can enhance IA risk assessment, as 3D-CAWE has a strong association with symptomatic presentation (OR 6.1, CI 2.1–19.5).

Despite the development of several scoring systems (Table 4), determining which IAs are at higher risk of becoming symptomatic is challenging. The PHASES score is the most recognized tool for predicting IA rupture, although it is not widely used in clinical practice [3]. It was created by pooling data from six prospective studies on cumulative rupture. Patient populations from these studies included patients from North America, Europe, and Japan, and it has not been validated in other populations, such as Chinese patients. This limitation likely contributed to its modest AUC of 0.64 in our study, where two-thirds of the cohort were Chinese. Other study with 1270 Chinese patients has similarly shown low performance of PHASES (AUC of 0.77) to predict IA growth and rupture [19]. Although our study did not analyze rupture risk, we used PHASES as the reference because it remains the most widely used clinical scoring system for IA to guide management decisions. Alternative scoring systems, such as ELAPSS [20] (Earlier SAH, location, age, population, size, and shape), ISUIA [21] (Earlier SAH, size, location), JRCCA [11] (Japanese Risk Score for Cerebral Aneurysm Rupture), and the Rotterdam score [22], incorporate morphological aneurysm features but are not consistently used in clinical practice.

**Table 4:** Scoring systems for intracranial aneurysm risk assessment

Score	Outcome	Variables
PHASES [3]	Rupture	Population, hypertension, age > 70, aneurysm size, earlier SAH and aneurysm location.
ELAPSS [19]	Growth	Population, age > 60, aneurysm size, earlier SAH, aneurysm location and shape.
ISUIA [20]	Rupture	Aneurysm size and location, and earlier SAH.
JRCCA [11]	Rupture	Hypertension, age > 50, aneurysm size, earlier SAH, aneurysm location and shape.
Rotterdam [21]	Rupture	Hypertension, age > 60, aneurysm size, aneurysm location and shape.

Abbreviations: *PHASES* = Population, Hypertension, Age, Size, Earlier subarachnoid hemorrhage, and Site; *ELAPSS* = Earlier SAH, location, age, population, size, and shape; *ISUIA* = International Study of Unruptured Intracranial Aneurysms; *JRCCA* = Japanese Risk Score for Cerebral Aneurysm Rupture.

Several of these scoring systems do not include factors that have been associated with increased risk of aneurysm rupture, such as smoking, female sex and irregular aneurysm morphology. In contrast, they include variables such as hypertension, which sometimes is already controlled with the use of medications. In our study, hypertension was not selected as a predictor in any of the top five performing models. Irregular shape and high-risk location (ACOM, PCOM and posterior circulation) were selected in the fourth and fifth top performing models but they were not independently associated with symptomatic presentation. AWE has shown potential in predicting aneurysm growth and rupture in prospective studies. A recent multicentric prospective study of 559 IAs found that 6.7% of IAs with AWE ruptured, compared to 2.5% of those without AWE (adjusted hazard ratio 1.4, 95% CI 0.5–3.7), after a median follow-up of 1.2 years [23]. Furthermore, a meta-analysis including 12 studies and 1761 IAs found that AWE was a predictor of rupture (prevalence ratio 11.47, 95% CI 4.05–32.46) and interval growth (prevalence ratio 4.62, 95% CI 2.85–7.49) [24]. These findings underscore the potential of AWE as a valuable neuroimaging biomarker for assessing IA rupture risk.

Despite the association between AWE and rupture risk, the use of heterogeneous assessment techniques has limited the widespread implementation of AWE evaluation in clinical practice [24]. Most prior studies used a subjective adjudication of AWE, which has shown high sensitivity, but inconsistent specificity. Recently, some authors have performed quantitative analyses of AWE, including the contrast ratio of the aneurysm wall to the pituitary stalk [25], the enhancement ratio (maximum signal intensity minus pre-contrast signal intensity, divided by pre-contrast signal intensity) [26], and the wall enhancement index (post-contrast signal intensity minus pre-contrast signal intensity, adjusted for white matter signal intensity) [27]. However, these methods rely on 2D measurements, which are prone to human error and bias due to the manual selection of regions of interest [24, 28]. To address these limitations, a 3D AWE objective quantification technique has been developed, which generates 3D color maps of Gd enhancement for more objective analysis. The advantage of 3D voxel-wise mapping is that it provides more accurate quantification by averaging the signal intensity across the entire wall surface and thickness. Additionally, 3D heatmaps visualization facilitates easier identification of contamination from adjacent structures. In this study, the identification of IA symptomatic presentation was enhanced in this study by incorporating 3D-CAWE to a comprehensive score that included clinical data and morphological aneurysm metrics.

Previous studies have also evaluated the performance of AWE in predicting IA rupture, symptomatic presentation, or growth, yielding promising but limited results. The “SAD score”, which combined IA location, AWE grading which was subjectively categorized as strong, faint or absent, and irregular IA shape, achieved an AUC of 0.88 [29]. However, this study was limited by a small retrospective dataset of 25 ruptured and 116 unruptured IAs, as well as the subjective adjudication of AWE. Fu et al. analyzed 100 unruptured IAs, with 28 symptomatic cases, and reported that wall permeability index and subjective AWE achieved an AUC of 0.81 for differentiating symptomatic from asymptomatic IAs [27]. In their study AWE patterns were subjectively adjudicated as absent, focal or circumferential wall enhancement. Similarly, Wang et al. reported that the enhancement ratio achieved an AUC of 0.90 for classifying symptomatic presentation of IAs, in a sample of 80 patients [30]. AWE in this study was assessed by sampling regions of interest along the aneurysm wall, and was subjectively classified as no enhancement, partial or entire wall enhancement. Despite their contributions, these studies share significant limitations, including reliance on subjective assessments of AWE and the manual selection of regions of interest for analysis, which introduces variability and limits reproducibility.

This study suggests that 3D-CAWE may serve as an adjunct to clinical criteria to triage the risk of aneurysms becoming symptomatic. However, several limitations outlined below should be accounted in future studies. First, the cross-sectional design of this study limits interpretation regarding future rupture risk and generalizability of the results. Our model was intended to predict symptomatic presentation at the time of imaging. We used symptomatic presentation as a surrogate marker of high-risk IAs. The lack of longitudinal data limits our ability to assess the temporal evolution of AWE. Future studies should incorporate serial imaging to assess temporal changes in AWE and incorporate time-to-event analyses to better understand how AWE relates to the timing of IA growth or rupture. Second, we did not perform internal or external validation of the model to ensure consistent performance outside the training dataset. External validation on an independent prospectively collected cohort is essential to confirm generalizability before clinical adoption. Third, the composite outcome included both ruptured and unruptured symptomatic cases, with the aim of reflecting a continuum of aneurysm instability driven by shared pathophysiology of inflammation and degenerative wall changes. However, some characteristics of the ruptured aneurysm wall may not reflect the pre-rupture status. For example, SAH surrounding the wall might produce enhancement artifacts. Fourth, our cohort might exhibit selection bias as high-resolution MRI is not standard of care by all providers in each institution, and therefore our cohort does not represent the total volume of patients who presented during the five-year enrollment period. Additionally, 29% of aneurysms were excluded due to suboptimal image quality, inability to identify the aneurysm wall or sinus wall enhancement contamination. The 3D-CAWE analysis of IAs located near enhancing structures, such as the cavernous sinus require meticulous voxel selection during image analysis to accurately isolate the aneurysm wall. Fifth, MRI acquisition parameters across participating centers were not identical. No signal-harmonization techniques were applied beyond corpus callosum normalization. However, this effect is likely minimal, as median 3D-CAWE values were similar across the Iowa, Changhai, and Renji cohorts (0.72, 0.71, and 0.70, respectively; *p* = 0.372). Future multicenter studies should incorporate fully standardized acquisition protocols and formal harmonization strategies to minimize scanner-related variability. Finally, the ICC between manual segmentations was 0.82. Given the small voxel size (1.08 mm³), small differences in wall segmentations were expected between raters. Future innovations, including the application of machine learning algorithms, hold the potential to automate IA segmentation and streamline the analytical process, thereby enhancing efficiency and reproducibility.

## Conclusions

In a large, international multi-center cohort of IAs assessed with high-resolution MRI, increased AWE was significantly associated with symptomatic presentation. The best performing model for estimating IA presentation incorporated clinical information, morphological aneurysm data and AWE metrics, underscoring the value of a comprehensive approach. Future longitudinal studies are essential to develop comprehensive predictive models incorporating advanced 3D AWE quantification to better assess the risk of IAs becoming symptomatic.

## Supplementary Information

Below is the link to the electronic supplementary material.

Supplementary Material 1

## Data Availability

No datasets were generated or analysed during the current study.
