# Supplementary material for: Analysis of symptomatic brain aneurysms with three-dimensional aneurysm wall enhancement
Source: Neurosurg Rev. 2026 Jan 24;49(1):159. doi: 10.1007/s10143-025-04120-w (PMC12831663; doi:10.1007/s10143-025-04120-w)
Supplement: Supplementary file 1 — Supplementary Material 1 [file 10143_2025_4120_MOESM1_ESM.docx]

**SUPPLEMENTARY MATERIAL**

Supplementary table 1. Subgroup analysis: unruptured versus ruptured

| Characteristic^1^, N = 387 | OR | 95% CI | p-value |
| --- | --- | --- | --- |
| Outcome: unruptured (n=60) | | | |
| Age | 0.96 | 0.93 – 0.98 | <0.001 |
| Size ratio | 1.7 | 1.4 - 2.1 | <0.001 |
| 3D-CAWE | 2.4 | 0.7 – 7.3 | 0.128 |
| Outcome: rupture (n=13) | | | |
| Smoking | 8.7 | 2.6 – 31.6 | <0.001 |
| Irregular | 4 | 1.1 – 14.3 | 0.028 |
| 3D-CAWE | 10.5 | 2.4 – 50.2 | 0.002 |

*^1^*Predictors were selected by all-subsets regression.

*Abbreviations: 3D-CAWE: three-dimensional circumferential aneurysm wall enhancement.*

Supplementary table 2. Comparison of the top five models from all-subsets logistic regression

| Characteristic^1^, N = 387 | OR | 95% CI | p-value |
| --- | --- | --- | --- |
| *Top 2 performing model AIC 320* | | | |
| Age | 0.95 | 0.94 - 0.98 | <0.001 |
| Size ratio | 1.6 | 1.3 - 1.9 | <0.001 |
| 3D-CAWE | 5.6 | 2 - 15.6 | <0.001 |
| *Top 3 performing model AIC 320* | | | |
| Age | 0.96 | 0.94 - 0.98 | <0.001 |
| Smoking | 2.1 | 1.1 - 4.3 | 0.041 |
| Size | 1.1 | 0.9 - 1.1 | 0.450 |
| Size ratio | 1.4 | 1.1 - 1.9 | 0.009 |
| 3D-CAWE | 5.6 | 1.8 - 14.8 | 0.002 |
| *Top 4 performing model AIC 321* | | | |
| Age | 0.96 | 0.93 - 0.98 | <0.001 |
| Smoking | 1.9 | 0.9 - 3.7 | 0.060 |
| Irregular | 1.2 | 0.6 - 2.3 | 0.570 |
| Size ratio | 1.5 | 1.3 - 1.9 | <0.001 |
| 3D-CAWE | 5.7 | 2.1 – 16.4 | <0.001 |
| *Top 5 performing model AIC 321* | | | |
| Age | 0.96 | 0.93 - 0.98 | <0.001 |
| Smoking | 1.9 | 0.9 - 3.7 | 0.060 |
| High risk location  (ACOM, PCOM  or posterior circulation) | 1.1 | 0.6 - 2.1 | 0.666 |
| Size ratio | 1.6 | 1.3 - 1.9 | <0.001 |
| 3D-CAWE | 5.7 | 2.1 – 16.3 | <0.001 |

*^1^*Predictors were selected by all-subsets regression.

*Abbreviations: ACOM: anterior communicating artery aneurysm; PCOM: posterior communicating artery aneurysm; 3D-CAWE: three-dimensional circumferential aneurysm wall enhancement.*
